# Facilitators, barriers, and changes in POCUS use: longitudinal follow-up after participation in a national point-of-care ultrasound training course in Japan

**DOI:** 10.1186/s13089-024-00384-3

**Published:** 2024-07-08

**Authors:** Toru Yamada, Nilam J. Soni, Taro Minami, Yuka Kitano, Shumpei Yoshino, Suguru Mabuchi, Masayoshi Hashimoto

**Affiliations:** 1 Department of General Medicine, Graduate School of Medical and Dental Sciences, Tokyo Medical and Dental University, Bunkyo-Ku, Tokyo, 113-8510 Japan; 2 Section of Hospital Medicine, South Texas Veterans Health Care System, San Antonio, Texas USA; 3 Division of Pulmonary Diseases and Critical Care Medicine, Joe R. and Teresa Lozano Long School of Medicine, University of Texas Health San Antonio, San Antonio, Texas USA; 4 Division of Hospital Medicine, Joe R. Teresa Lozano Long School of Medicine, University of Texas Health San Antonio, San Antonio, Texas USA; 5 Medicine Division of Pulmonary, Critical Care, and Sleep Medicine, The Warren Alpert Medical School of Brown University, Providence, RI USA; 6 Medicine Division of Pulmonary, Critical Care, and Sleep Medicine, Care New England Health System, Providence, RI USA; 7 Emergency and Critical Care Medicine, School of Medicine, St. Marianna University, Kawasaki, Kanagawa Japan; 8 General Internal Medicine, Iizuka Hospital, Iizuka, Fukuoka Japan

**Keywords:** Point of care Ultrasound, POCUS, Training, Barriers, Facilitators, Longitudinal follow Up

## Abstract

**Background:**

POCUS training courses are effective at improving knowledge and skills, but few studies have followed learners longitudinally post-course to understand facilitators, barriers, and changes in POCUS use in clinical practice. We conducted a prospective observational study of physicians who attended 11 standardized POCUS training courses between 2017 and 2019 in Japan. Physicians who attended a standardized POCUS course were surveyed about their current frequency of POCUS use of the heart, lung, abdomen, and lower extremity veins, and perceived barriers and facilitators to POCUS use in clinical practice.

**Results:**

Data were analyzed from 112 completed surveys (response rate = 20%). A majority of responding physicians were faculty (77%) in internal medicine (69%) affiliated with community hospitals (55%). The mean delay between course attendance and survey response was 50.3 months. A significant increase in POCUS use from < 1 to ≥ 1 time per week was seen for all organ systems after 50 months post-course (p < 0.01). Approximately half of course participants reported an increase in the frequency of cardiac (61%), lung (53%), vascular (44%), and abdominal (50%) ultrasound use. General facilitators of POCUS use were easy access to ultrasound machines (63%), having a colleague with whom to learn POCUS (47%), and adequate departmental support (46%). General barriers included lack of opportunities for POCUS training (47%), poor access to ultrasound machines (38%), and limited time for POCUS training (33%). In the group with increased POCUS usage, specific facilitators reported were enhanced POCUS knowledge, improved image acquisition skills, and greater self-confidence in performing POCUS. Conversely, the group without increased POCUS usage reported lack of supervising physicians, low confidence, and insufficient training opportunities as specific barriers.

**Conclusions:**

Approximately half of physicians reported an increase in cardiac, lung, vascular, and abdominal POCUS use > 4 years after attending a POCUS training course. In addition to improving access to ultrasound machines and training opportunities, a supportive local clinical environment, including colleagues to share experiences in learning POCUS and local experts to supervise scanning, is important to foster ongoing POCUS practice and implementation into clinical practice.

**Supplementary Information:**

The online version contains supplementary material available at 10.1186/s13089-024-00384-3.

## Background

Point-of-care Ultrasound (POCUS) is an effective bedside diagnostic tool that has been shown to improve patient outcomes in recent years [1]. As more physicians learn about POCUS, its usage continues to expand across nearly all clinical specialties and settings, including acute care hospitals, primary care clinics, and resource-limited and rural settings [2–4].

Despite its potential advantages, widespread adoption of POCUS use has been slow, with reports suggesting that only about 10% of general physicians use POCUS [4]. Several studies have revealed important barriers to POCUS use [3, 5–9]. Currently, lack of training in POCUS has been reported as a top barrier to POCUS by several specialties, including lack of trained faculty to teach medical students and residents [5, 9–12].

To address the training gap, POCUS training courses have been developed in several specialties for physicians in-practice, often in collaboration with large national specialty societies. These POCUS training courses are typically 2–3 days in duration and have been shown to improve learners’ immediate post-course knowledge and skills [13–15]. However, despite the immediate improvements post-course, few studies have reported long-term retention of POCUS knowledge and skills or changes in frequency of POCUS use in clinical practice [15–18]. Furthermore, little is known about the barriers and facilitators to ongoing POCUS use after participating in a POCUS training course.

To evaluate the impact of participating in a POCUS training course on clinical practice, we surveyed participants of a standardized POCUS course on their frequency of POCUS use post-course and assessed barriers and facilitators to better understand implementation of POCUS use in clinical practice.

## Methods

### Study design

A prospective observational study was conducted as a follow-up to a prior study evaluating the educational effectiveness of 11 hands-on POCUS training courses held between 2017 and 2019 [19]. The course curriculum was modeled after POCUS training courses developed by the Society of Hospital Medicine and the American College of Chest Physicians. The courses were accredited by the Japanese Association for Acute Medicine and the Japanese Society of Hospital General Medicine. The course spanned two days and consisted of five lectures (Focused Cardiac Ultrasound (FOCUS); lung, abdominal, and lower extremity venous ultrasound; shock assessment; and multi-system cases) and six hands-on sessions [FOCUS (3 sessions) followed by lower extremity venous, lung, and abdominal ultrasound sessions]. A detailed outline of the course content is shown in Additional File 1.

In January 2023, a follow-up email survey was sent to all participants of 11 past POCUS courses who consented to participate in the study and completed the entire two-day training course. This study adhered to the guidelines of the Declaration of Helsinki and received authorization from the Institutional Review Boards of both the Tokyo Bay Urayasu Ichikawa Medical Center (protocol number 265) and the Tokyo Medical and Dental University (protocol number M2019-085). All participants of the study provided written informed consent.

### Study participants

Study participants were physicians and senior medical students in their 5th or 6th years who had attended a past POCUS course. In Japan, after completing 6 years of medical school following high school graduation, all physicians participate in a mandatory 2-year junior residency with rotations in internal medicine, surgery, emergency medicine, and other specialties. Afterward, physicians become senior residents and can undertake a 3-year specialty training program in their chosen field, such as internal medicine, surgery, or emergency medicine. After completing their senior residency, physicians must pass a specialty board examination to become specialists in their respective fields. Physician specialists were defined as faculty in the current study.

### Assessment tools and data collection

Course participants completed a pre-course survey to collect background information on their profession, affiliated institution(s), department, and baseline use of common POCUS applications of the heart, lung, abdomen, and lower extremity veins [19]. The post-course survey questions were developed based on a combination of published similar surveys and discussion among our authors of the most relevant data to collect [5, 9, 20]. The post-course survey queried participants’ clinical rank, institution(s) and department affiliation(s), dates of course attendance, frequency of POCUS use for each organ system, reasons for any changes in frequency in POCUS use, and perceived barriers and facilitators to POCUS use. The post-course survey used in this study is shown in Additional File 2.

### Data analysis

Pre- and post-course frequencies of POCUS usage were compared using the Fisher's exact test. Cardiac, lung, vascular, and abdominal ultrasound usage were evaluated separately and categorized for comparison as either ≥ 1 or < 1 time per week. A cut-off of 1 was used to determine any POCUS use since previous studies have demonstrated low usage of POCUS in Japan. [19] Groups that increased their overall POCUS usage were compared with those that did not based on reported barriers and facilitators. Subgroup analyses by facility and department for barriers and facilitators were also conducted. Data analyses were performed using STATA 17.0 (StataCorp LLC, College Station, TX, USA). A p-value of less than 0.05 was considered statistically significant.

## Results

Surveys were sent to 571 course participants who came from 43 of the 47 prefectures of Japan. A total of 134 survey responses were received, and data were analyzed from 112 surveys with complete answers to all questions (response rate = 20%). Course participants' characteristics at the time of the survey are shown in Table 1. Among the 112 responding course participants, a majority were male faculty who specialized in internal medicine and were affiliated with community hospitals.

**Table 1:** Participants' characteristics (n = 112)

Characteristic	n	(%)
Sex		
Male	83	(74)
Female	29	(26)
Practice Setting		
Private clinic	12	(11)
Community hospital	62	(55)
University hospital	32	(29)
Other	6	(5)
Clinical rank		
Junior resident^1^	1	(1)
Senior resident^2^	25	(22)
Faculty^3^	86	(77)
Medical student^4^	0	(0)
Specialty		
Physician: IM/GM and IM Subspecialties	78	(69)
Physician: Critical care, emergency medicine	12	(11)
Physician: Family medicine	4	(4)
Physician: Junior resident	1	(1)
Physician: Other	17	(15)
Medical student^4^	0	(0)
Number of months from course participation to survey	52 (39–58)	Median (IQR)

^1^Junior resident: postgraduate year 1 and 2 physicians who were under the 2-year National Obligatory Initial Postgraduate Clinical Training Program

^2^Physicians with specialty training after completion of junior residency

^3^Physicians after completion of senior residency

^4^Fifth and sixth year medical students

*IM* Internal medicine, *GM* General medicine, *SD* Standard deviation, *IQR* Interquartile range

**Fig. 1:**
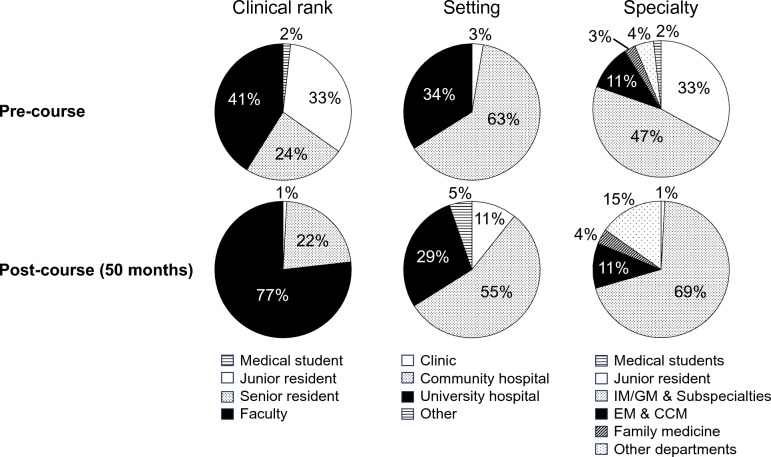
Change in Course Participants’ Characteristics from Pre-course to 50-months Post-course. *IM* internal medicine, *GM* general medicine, *EM* emergency medicine, *CCM* critical care medicine

The mean time between course attendance and survey response was 50.3 months (minimum 37, maximum 64 months). The course participants’ characteristics changed from the time of course attendance to the time of the post-course survey (Fig. 1). The clinical rank distribution shifted as junior residents became faculty and several physicians completed training in internal medicine & its subspecialties. Given the lapse of approximately four years from course attendance to post-course survey, only one respondent (1%) was a junior resident, and no medical students were among the respondents at the time of the post-course survey.

### Frequency of POCUS use

**Fig. 2:**
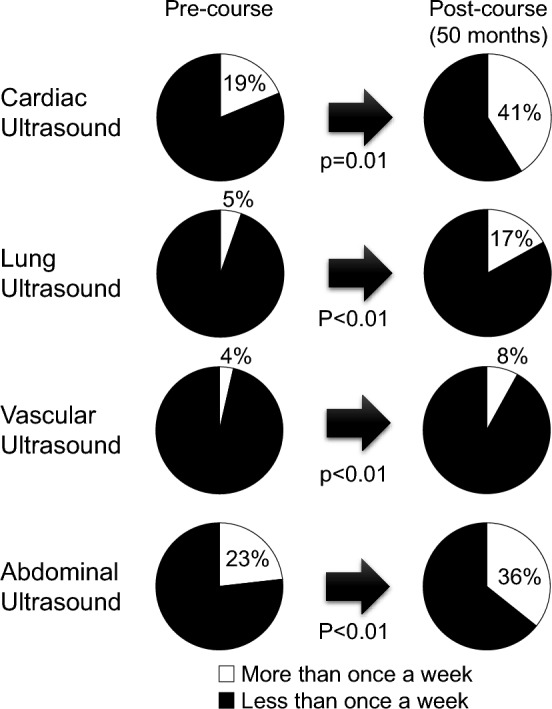
Change in POCUS Use per Organ System from Pre-course to 50-months Post-course. *POCUS* point of care ultrasound

Approximately half of the course participants reported an increase in the frequency of cardiac (61%), lung (53%), vascular (44%), and abdominal (50%) ultrasound use after 50 months post-course. When comparing the reported frequencies of POCUS use pre- and 50 months post-course, the proportion of participants who increased their frequency of use (for example, from “Never” to “Less than once a month” or from “Less than once a month” to “Two or three times a month”) was 38% for cardiac, 47% for lung, 45% for vascular, and 31% for abdominal ultrasound. A statistically significant increase in POCUS use from < 1 to ≥ 1 time per week was seen for all organ systems from pre-course to 50 months post-course (Fig. 2). The greatest increases were seen in cardiac and abdominal ultrasound usage.

### Barriers and facilitators of POCUS use

**Fig. 3:**
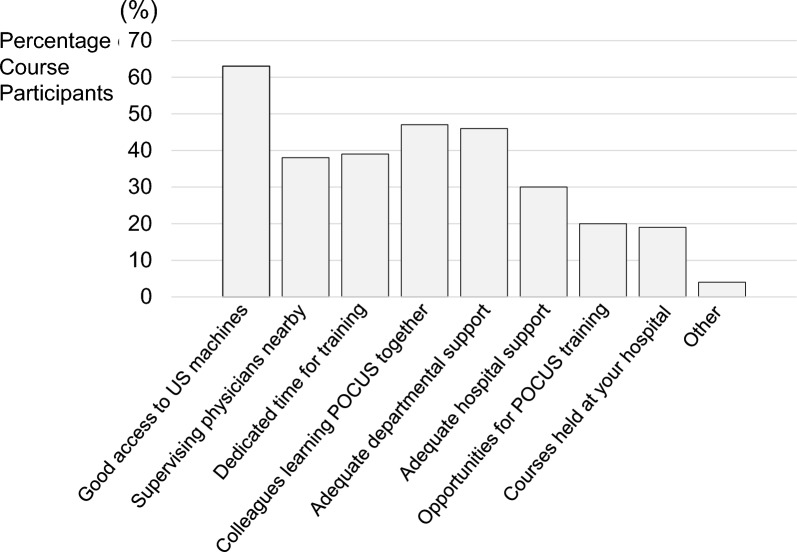
General Facilitators to POCUS Use. *POCUS* point of care ultrasound, *US* ultrasonography

**Fig. 4:**
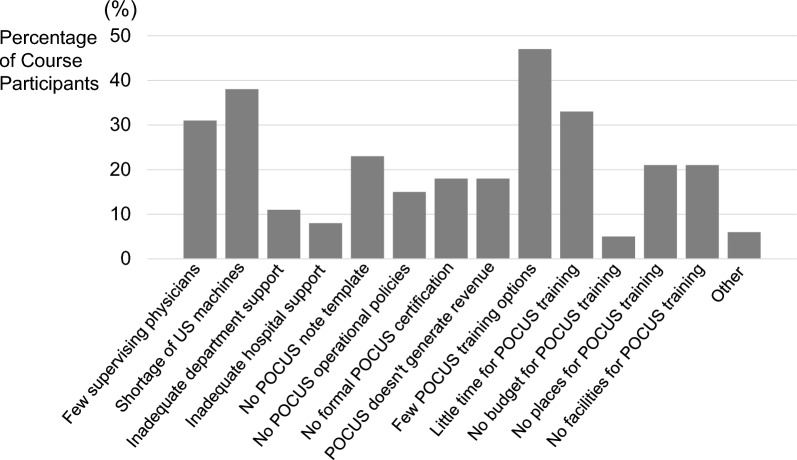
General barriers to POCUS Use. *POCUS* point of care ultrasound, *US* ultrasonography

All course participants were asked about general facilitators and barriers to POCUS use (Figs. 3 and 4). The most common facilitators to POCUS use were ready access to ultrasound machines (63%), having a colleague to learn POCUS together (47%), and adequate departmental support (46%). The most common barriers reported were availability of POCUS training courses (47%), access to ultrasound machines (38%), and limited time for POCUS training (33%). An analysis of the top three facilitators and barriers did not reveal any significant differences by specialty or setting.

We compared the facilitators and barriers reported by groups that increased versus did not increase POCUS usage. The only facilitator that was reported significantly more by groups that increased usage was having a colleague who was learning POCUS simultaneously (p = 0.03) (Table 2).

**Table 2:** General Facilitators and Barriers by Physicians that Increased vs. Did Not Increase POCUS Usage Post-course

General facilitators to performing POCUS	All participants n = 112 (%)	POCUS Usage increased n = 74 (%)	POCUS usage did not increased n = 38 (%)	*p* value
Good access to US machines	71 (63)	44 (59)	27 (71)	0.30
Supervising physicians are nearby	42 (38)	31 (42)	11 (29)	0.22
Training time secured	44 (39)	29 (26)	15 (38)	1.00
Colleagues learning POCUS together	53 (47)	41 (55)	12 (32)	0.03*
Adequate departmental support	52 (46)	38 (51)	14 (37)	0.17
Adequate hospital support	34 (30)	26 (35)	8 (21)	0.14
Many chances for POCUS training	22 (20)	17 (23)	5 (13)	0.32
Courses held at your hospital	21 (19)	17 (23)	4 (11)	0.13
Few supervising physicians	35 (31)	26 (35)	9 (24)	0.28
Shortage of US machines	43 (38)	26 (35)	17 (45)	0.41
Inadequate departmental support	12 (11)	10 (14)	2 (5)	0.22
Inadequate hospital support	9 (8)	7 (9)	2 (5)	0.72
No POCUS record format	26 (23)	18 (24)	8 (21)	0.82
No operation rules for POCUS	17 (15)	12 (16)	5 (13)	0.79
No formal POCUS certification	20 (18)	14 (19)	6 (16)	0.80
POCUS doesn't generate revenue	20 (18)	14 (19)	6 (16)	0.80
Few POCUS training chances	53 (47)	39 (53)	14 (37)	0.16
Little time for POCUS training	37 (33)	22 (30)	15 (39)	0.40
No budget for POCUS training	6 (5)	4 (5)	2 (5)	1.00
No places for POCUS training	23 (21)	19 (26)	4 (11)	0.08
No facilities for POCUS training	23 (21)	16 (22)	7 (18)	0.81

*US* Ultrasound, *POCUS* Point of care ultrasound, *p < 0.05

**Fig. 5:**
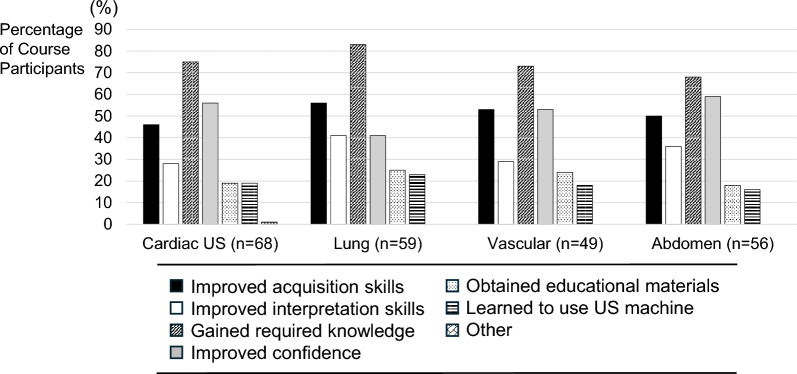
Specific Facilitators Associated with Increased POCUS use. *US* ultrasonography

**Fig. 6:**
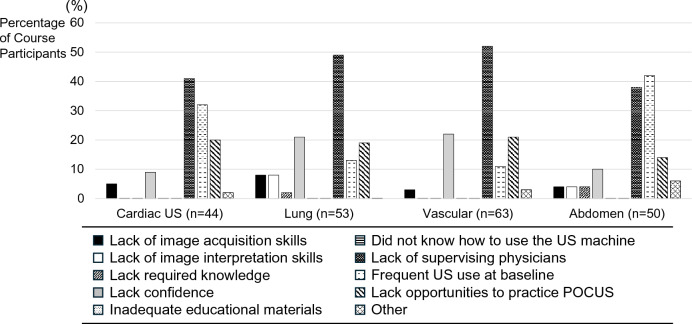
Specific Barriers Associated with Not Increasing POCUS use. *POCUS* point of care ultrasound, *US* ultrasonography

Course participants who reported an increase in POCUS use post-course were asked about specific facilitators of POCUS use (Fig. 5), whereas those who reported no increase in POCUS use post-course were asked about specific barriers to POCUS use (Fig. 6). The most common facilitators reported across all POCUS applications were improvements in POCUS knowledge, image acquisition skills, and self-confidence in performing POCUS examinations. The most common barriers reported were lack of supervising physicians to provide guidance, lack of confidence, and lack of opportunities to practice POCUS applications. For lung and vascular ultrasound, a significant reason for not increasing frequency of use was a lack of confidence. In contrast, for cardiac and abdominal ultrasound, a substantial proportion of course participants were already performing these specific POCUS applications greater than once per week pre- and post-course, and therefore, they were included in the subgroup reporting no increase in POCUS use post-course. None of the course participants reported insufficient educational materials or difficulty in using the ultrasound machine as barriers.

## Discussion

We conducted a longitudinal study to evaluate changes in POCUS use reported by physicians 50 months after participating in a standardized, hands-on POCUS training course. A significant increase in the performance of cardiac, lung, vascular, and abdominal POCUS exams was seen and the proportion of physicians performing ≥ 1 POCUS exams of different organ systems per week. Additionally, we have revealed important facilitators and barriers to continued POCUS use post-course that can guide POCUS implementation efforts.

Past studies have demonstrated immediate improvements in POCUS knowledge and skills after participating in a hands-on POCUS course [13, 14, 21], but few studies have followed course participants longitudinally to determine actual POCUS use in clinical care [15–18]. Two small studies (n = 17–20) from limited-resource settings in Africa reported retention of POCUS knowledge and skills by physicians in-practice after 9–12 months and increased POCUS use post-course; however, details about increased use were not provided [18, 22]. A study of physicians practicing in the Department of Veterans Affairs demonstrated sustained improvement of both POCUS knowledge and skills at 8 months post-course, and course participants reported a significant increase in multi-system POCUS use (heart, lungs, abdomen, and vascular access) in clinical care from pre-course to 8 months post-course [15]. To assess how physicians in-practice use POCUS beyond 12 months post-course, we surveyed physicians in-practice longitudinally after > 3 years to determine if increased POCUS use was sustained in clinical practice and better understand factors that may be associated with sustained increased POCUS use. A statistically significant increase in POCUS use to ≥ 1 time per week was seen for all organ systems. Specifically, the frequency of cardiac, lung, vascular, and abdominal ultrasound use increased by approximately half of physicians at 50 months post-course. However, the increase in lung and vascular ultrasound use ≥ 1 time per week was relatively low (17% and 8%, respectively) compared to cardiac and abdominal ultrasound (41% and 36%, respectively). The main barriers associated with not increasing lung and vascular ultrasound use were “lack of supervising physicians” and “lack of confidence.” We speculate that physicians were unable to obtain adequate supervised practice to gain comfort and competence in performing lung and vascular ultrasound exams after returning to their home institutions.

Barriers and facilitators to POCUS use in different specialties and settings have been described in several studies [2–11, 23]. In general, lack of access to ultrasound equipment and lack of POCUS training, including availability and time for training, have been the two most common barriers reported to start using POCUS. However, little is known about barriers and facilitators to sustained POCUS use after physicians have received training. Based on our literature review, our study is the first to evaluate provider- and facility-level facilitators and barriers associated with increasing or not increasing POCUS use in clinical practice > 3 years post-course. Having a colleague with whom to learn POCUS was the only facilitator that was shown to be significantly associated with sustained increased POCUS usage. On the contrary, among physicians who did not increase POCUS use post-course, the most frequently reported specific barrier was lack of supervising physicians to provide guidance.

The implications of our study are important for hospitals and health systems seeking to standardize and implement POCUS use systemwide. First, for physicians in-practice, brief hands-on POCUS training courses of 2–3 days have been shown to increase clinical POCUS use for 6–12 months post-course, and our study adds that increased clinical POCUS use is sustained > 3 years post-course among a significant proportion of physicians. Second, the most common barriers to starting POCUS use, namely lack of access to ultrasound equipment and lack of training, are different from the barriers to sustaining its use. A supportive clinical environment with readily available POCUS experts who can provide ongoing supervision and adequate departmental and hospital support are critical for long-term success of POCUS implementation [24]. Third, since having a colleague with whom to pursue POCUS training together facilitated long-term clinical POCUS use, it is plausible that organizing training cohorts may be a more effective approach to deploy systemwide POCUS training which can be trialed in future training studies.

We recognize our study has limitations. Most important, our post-course survey response rate was 20%, and the possibility of sample bias due to the low response rates cannot be ruled out. Though we had a relatively low post-course response rate, the absolute number of completed surveys was 112 which is higher than most similar studies. Of note, the pre-course survey response rate was 100% because answering the pre-course survey was a mandatory part of the application process. Additionally, we collected self-reported data that may not accurately reflect actual clinical practice. Finally, we were unable repeat POCUS knowledge and skills testing to assess retention because course participants came from 43 of 47 prefectures across Japan and coordinating logistics for testing was not feasible.

## Conclusions

Approximately half of physicians reported an increase in cardiac, lung, vascular, and abdominal POCUS use > 4 years after attending a POCUS training course. Our findings confirmed well known barriers to POCUS use, including lack of access to ultrasound machines and training opportunities and revealed the importance of a supportive local clinical environment, including having colleagues with whom to learn POCUS collaboratively, local POCUS experts available to supervise scanning, and departmental support for POCUS implementation. Hospitals and health systems seeking to implement standardized POCUS use shall invest in developing supportive clinical environments that foster ongoing POCUS practice and use in patient care.

### Supplementary Information

Additional file 1: Point of care Ultrasound Course Contents

Additional file 2: Point of care Ultrasound Post-course Survey Questions

## Data Availability

The datasets used and/or analyzed during the current study are available from the corresponding author upon reasonable request.

## Abbreviations

POCUS: Point-of-care Ultrasound
FOCUS: Focused cardiac ultrasound
LMIC: Low-and middle-income countries
US: Ultrasonography
IM: Internal medicine
GM: General medicine
SD: Standard deviation
VA: Veterans Affairs
